# Medical Opinion and Movement

**Published:** 1911-10-14

**Authors:** 


### MEDICAL OPINION AND MOVEMENT.

### Value of the Albumin Reaction with Sputum.

Since the original memoir of Roger and Valensi, showing the importance of looking for albumin in the sputum, a number of articles on the same subject have appeared; among the latest is that of Lesieur and Privey, published in *Paris Médical*. Their technique is as follows: The sputum is collected in a dry bottle and, as far as possible, is kept free from saliva. To the sputum is added an equal volume of 7 per 1,000 saline solution, the whole being then agitated with a glass rod and filtered. To the filtrate four or five drops of acetic acid are added to precipitate mucin and fibrin, the result being filtered again, and the usual tests for albumin applied to the second filtrate. The nitric acid test is the most useful, and when traces of albumin are present a bluish liquid will be formed at the junction of the two fluids. A moderate reaction will show a cloudy ring, and an intense reaction a still denser ring at the same junction. In all established cases of phthisis investigated by the authors, examination of the sputum for albumin gave a positive result. The reaction is, however, of greatest service in doubtful cases. Out of one hundred and sixty-seven, the authors have obtained one hundred and thirty-seven positive results. In certain congestive states of the lung, especially in typhoid cases, the reaction varies from time to time. In pneumonia it is positive, but disappears during convalescence. Apart from pulmonary conditions the reaction may be positive in cardiac and renal conditions without the presence of pulmonary lesions. Much of the value of the reaction is lost, therefore, when dealing with senile cases.

### New Method of Skin Grafting.

In the *China Medical Journal* Dr. C. H. Barlow describes a method of skin grafting which, though he disclaims any originality for it, is novel as far as Great Britain is concerned. He aims at securing for the denuded surface a sprinkling of the cells of the *stratum germinativum* of the skin, which lie next to the corium. These cells have, he says, a most amazing vitality, and can even be kept alive in normal salt solution for many days or grown upon culture media. Moreover, each cell reproduces itself in favourable conditions in from one to eight hours, so that the grafts spread rapidly. In order to get these proliferating cells, the superficial layers must first be got out of the way; and the author's method is extremely simple. He uses an ordinary stiff fibre brush, such as is used for cleaning vegetables, or a stiff hand-brush. He scrubs the area adjacent to the denuded surface till it is thoroughly reddened and denuded of the superficial layers of non-proliferating cells. He then scrubs the denuded surface, if granulations are present, until it is clean and bleeding; if it is fresh it need not be scrubbed. Without waiting for bleeding to cease, he scrubs from the prepared sound surface down over the denuded area with a strong even stroke, stopping the brush with a slight tamping movement. This carries the fresh cells down over the denuded area and implants them. No especial care is needed subsequently beyond simple dressings, and the results are said to be excellent. Though somewhat painful, the process does not require anæsthesia.

### Phthisis and Nasal Obstruction.

Some revolutionary ideas on treatment are advanced by Dr. C. A. Bucklin in the *Journal of Laryngology, Rhinology, and Otology*. He begins by postulating that pulmonary tuberculosis can be cured by curing the bronchitis which accompanies it. If by this he means that pulmonary tuberculosis begins around and in the small bronchioles, he is merely tautological; but if he refers to bronchitis not due to the tubercle bacillus, he ought to advance some proof or evidence of his statement. He goes on to claim that bronchitis can best be cured by resort to high altitudes, a not unreasonable view; and goes on to assert that by removal of certain parts of one or both lower turbinal bones the patient can be subjected to the atmospheric conditions prevailing at altitudes of 15,000 to 30,000 feet. Of this proposition again he offers no proof, and it is on the face of it so absurd that the rest of his argument must be regarded with some reserve. The rest of the treatment is forced diet of pure rich sterilised milk. Of this an adult man is made to take a tumblerful every thirty-six minutes during the daytime, ice cold. To the first and last pints of the milk are added 2 drachms of dilute hydrochloric acid, to assist digestion. Women receive the same food, but are allowed forty-five minute intervals instead of thirty-six. A teaspoonful of castor oil in black coffee is given every night at bedtime. It is stated that good results follow, but no statistics or illustrative cases are given.

### Shoulder-joint Dislocations.

In the *Annals of Surgery* Dr. Turner Thomas discusses the pathology of nerve troubles following dislocation of the shoulder joint. Such complications are usually explained by compression of the axillary nerves or by lesions of the spinal nerve roots. Various mechanical hypotheses have been conceived to explain these accidents. Dr. Thomas offers, however, a different explanation. Dislocations and simple sprains of the shoulder joints are generally produced with the arm in abduction. As a consequence, the antero-inferior parts of the capsule are subjected to special violence, so as in all probability to lead to tearing. This tearing may be accompanied by hæmorrhage, and in any case becomes the focus of a formation of fibrous tissue in the axilla, which may set up a perineuritis. Immobilisation, with the arm flexed to the side, aggravates the condition, and when the attempt is made to move the arm again, it is prevented by the axillary fibrous bands that have been formed in the meantime. Paralytic and painful nervous conditions are also likely to follow from the compression of the nerve trunks by these fibrous tissue formations. The author supports this view by several cases which have been cured by forcible abduction and breaking of these supposed adhesions on the antero-inferior surface of the capsule. This hypothesis might also be applied to explain certain birth paralyses, following traction on the arms, in which a sprain or subluxation of the shoulder joint might be accompanied by tearing of the internal part of the capsule. In the light of this hypothesis the treatment should be directed towards early movements of the joint and rapid absorption of any axillary hæmatoma.

### Intramuscular Injections.

Some years back Meltzer and Auer demonstrated by experiments on animals that the intramuscular injection of a substance is much more prompt in its effect than a subcutaneous injection, and is almost as rapid and effectual in its action as an intravenous injection. This rapid absorption in the muscles is explained by the rich capillary supply to the muscles and by the elevation of intramuscular pressure consequent on the injection, causing a more rapid penetration of the solution through the capillary walls into the general blood-stream. In the *Berliner Klinische Wochenschrift*, Meltzer returns to this subject and points out that these conditions are not so favourable in all the muscles of the body, and he is of opinion that the muscles of the buttocks are much inferior in this respect to the lumbar muscles. The lumbar muscles are denser and the muscular fibres finer and interwoven with comparatively little connective tissue, and the whole mass is embraced by two thick aponeuroses. On the other hand, the muscles of the buttocks consist of coarse fibres separated by much fatty and connective tissue, and their aponeuroses are loose, so that the injected fluid can easily escape into the cellular tissue and fails to exert that intramuscular pressure which is so important a factor for rapid absorption. Meltzer is of opinion that the accidents following injections of salvarsan into the buttocks—persistent pain, sciatica, paralysis of the peroneal nerve, thrombosis, etc.—are not the result of the intramuscular injection itself, but are due to the arsenical preparation spreading into the loose cellular tissue around the nerve trunks and blood vessels. On these grounds, therefore, he advocates intramuscular injections into the lumbar muscles. The method of procedure is as follows: The patient should lie down face downwards, so as to raise the lumbar region and make the lumbosacral muscles prominent. A line is then drawn from the most prominent point of the iliac crest to the spinous process of the third or fourth lumbar vertebra. The needle is introduced at the junction of the inner and middle thirds of this line. The needle should be inserted perpendicularly downwards towards the abdomen, till it reaches the aponeurosis, which is very resistant. Having penetrated this it should be carried towards the head parallel with the vertebral column and a little inwards. The needle must be sufficiently strong and should be at least eight to ten centimetres long. In obese subjects the layer of fat between the skin and the sacro-lumbar aponeurosis is often very thick. The author has carried out these injections in twelve patients and the results have been very satisfactory, the injections causing little or no pain.

### Tuberculosis and Syphilis.

Some of his impressions of the interrelation of these two infections are embodied in a lecture by Mr. D'Arcy Power, printed in the *Clinical Journal*. As clinical memoranda from an extensive experience they are worth quotation. Children with heredo-syphilis often show signs of tuberculous disease of the joints in the form of a "gummatous" synovitis, which does not improve beyond a certain point under treatment by antisyphilitic remedies. In young adults born of syphilitic parents an attack of pneumonia or other infective disease may be followed by signs of syphilitic inflammation. The syphilitic lesions thus manifested may become tuberculous and the patient may die of tuberculosis. Adults who acquire syphilis whilst they are suffering from active tubercle are liable to have any pre-existing tuberculous inflammation rendered much more active. It is to these persons that the generalisation applies—"The tuberculous bear syphilis badly." Old persons who acquired syphilis many years previously may be attacked by senile tuberculosis. In such patients the disease runs a more chronic course than usual, and there is less tendency to suppuration.

### Maternal Nursing in New York.

In some ways the difficulty of encouraging maternal nursing as opposed to bottle feeding is greater in America even than here. Notwithstanding the consensus of opinion in the medical profession that, with rare exceptions, every mother ought to and can nurse her child, there are still encountered far too often those who allow their patients to abandon the attempt on totally insufficient grounds. Some valuable work in the promotion of breast-feeding is being done in New York by a clinic established for that purpose, and in *Archives of Pediatrics* Dr. Schwarz gives some interesting figures from his records. Out of 1,500 patients, only thirty-six failed to feed their own babies for a month at least; eleven of these had tuberculosis and were therefore not allowed to nurse; six were in hospital for various diseases; six had inverted nipples; and only four out of the 1,500 failed because they really had no milk. This shows how rare complete agalactia is. For three months eighty-eight per cent. of the children were nursed, and a further four per cent. had mixed feeding; even at seven months but thirteen per cent. were having the bottle only. Other tables of great interest are included, bearing out familiar truths; it is remarkable that the mortality of the children of American-born mothers was nearly twice as great as that of those born of foreign mothers, a difference partly, but surely not wholly, explicable by the better percentage of breast-feeding among the latter.

